# Immune Regulation in T1D and T2D: Prospective Role of Foxp3+ Treg Cells in Disease Pathogenesis and Treatment

**DOI:** 10.3389/fendo.2013.00076

**Published:** 2013-06-25

**Authors:** Mara Kornete, Edward S. Mason, Ciriaco A. Piccirillo

**Affiliations:** ^1^Department of Microbiology and Immunology, McGill University, Montreal, QC, Canada; ^2^FOCIS Center of Excellence, Research Institute of the McGill University Health Center, Montreal, QC, Canada

**Keywords:** inflammation, obesity, Foxp3, Treg, diabetes, metabolic, immune regulation

## Abstract

There is increasing evidence that dysregulated immune responses play key roles in the pathogenesis and complications of type 1 but also type 2 diabetes. Indeed, chronic inflammation and autoimmunity, which are salient features of type 1 diabetes, are now believed to actively contribute to the pathogenesis of type 2 diabetes. The accumulation of activated innate and adaptive immune cells in various metabolic tissues results in the release of inflammatory mediators, which promote insulin resistance and β-cell damage. Moreover, these dysregulated immune responses can also mutually influence the prevalence of both type 1 and 2 diabetes. In this review article, we discuss the central role of immune responses in the patho-physiology and complications of type 1 and 2 diabetes, and provide evidence that regulation of these responses, particularly through the action of regulatory T cells, may be a possible therapeutic avenue for the treatment of these disease and their respective complications.

## The Pathogenesis of Type 1 Diabetes

**Figure 1:**
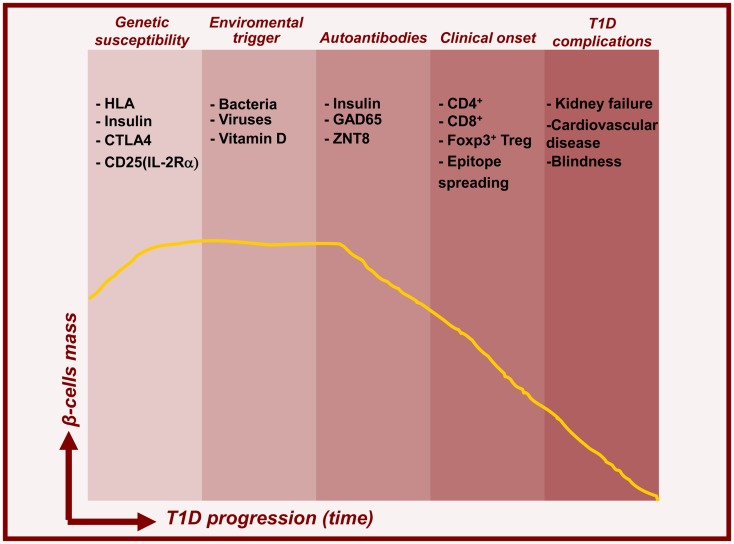
**Timelines for type 1 diabetes**. Model for temporal relationship between beta-cell mass decline and features of T1D pathogenesis. In addition to genetic predisposition, environmental triggers induce islet autoimmunity and beta-cell death leading to prediabetes and subsequent clinical onset and complications.

Type 1 diabetes (T1D) is a chronic autoimmune disease resulting from a T cell-dependent (both CD4^+^ and CD8^+^) destruction of the insulin-producing β-islets of Langerhans in the pancreas, leading to insulin deficiency and persistent hyperglycemia (Figure 1). Upon β-cells destruction, T1D patients lose blood glucose control, which provoke severe hyperglycemia. Even with current insulin replacement therapies secondary complications such as heart disease, blindness, and kidney failure may arise. Diagnosis is typically made early in life, with onset as young as 1 year of age and in most cases before the age of 18. The appearance of diabetes associated autoantibodies in the serum is the first detectable sign of emerging β-cell autoimmunity with over 90% of T1D patients testing positive for at least one at the time of diagnosis. Notable T1D auto-antigens identified include insulin, GAD65 (glutamic acid decarboxylase, 65 kDa isoform), IA2 (insulin auto-antigen 2), and zinc transporter 8 (ZNT8) (Sabbah et al., 1999; Orban et al., 2009). Several reports suggest that insulin is a primary auto-antigen for disease initiation. For example, elimination of pro-insulin or insulin completely abolished insulitis and T1D in NOD mice, while removal of another islet antigen, IGRP, did not show protective effect (Krishnamurthy et al., 2006). Both, genetic and poorly defined environmental factors act together to precipitate disease progression. Most studies confirm a global increase in incidence of T1D, particularly among young children. This likely reflects various environmental changes, although the impact of any individual exogenous factor has not yet been definitively proven. Pathogens such as viruses and bacteria, early exposure to cow’s milk, gluten, and meat preservatives and deficiency in dietary Vitamin D or omega 3 fatty acids have been proposed to contribute to the pathogenesis of T1D (Knip et al., 2005). Many epidemiological efforts have been made to understand the potential role of viruses in T1D pathogenesis. It is possible that viral antigenic mimicry could result in cross-reactive responses toward islet antigens. The striking sequence similarities between the 2C protein from coxsackievirus and GAD, a major auto-antigen in T1D, support this notion (Kaufman et al., 1992). Alternatively induction of a pro-inflammatory anti-viral response to infection, could activate innate immune cells, break tolerance, and initiate autoimmunity. It has also been established that in response to the viral infection, endocrine islet cells are able to produce pro-inflammatory cytokines, such as IL-8, IL-6, TNFα, and CXCL10 that could further trigger abnormal immune responses and T1D (Christen et al., 2003; Berg et al., 2006). The gut microbiota, via interaction with the host innate immune system, has been shown to modulate T1D onset (Chervonsky, 2010). For example, in NOD mice, T1D incidence dramatically decreases when mice are exposed to various microbial products. Similarly, the so called “hygiene hypothesis” suggests that the marked increase in T1D incidence in industrialized countries is related to reduced helminth burden therein (Mathis and Benoist, 2012).

Early studies indicate that multiple genes within human leukocyte antigen (HLA) on chromosome 6 are critical susceptibility loci for human autoimmune disease, including T1D. Two T1D associated haplotypes, namely DR4-DQ8 and DR3-DQ2, are present in 90% of children with the disease. Candidate gene studies identify insulin as a second important gene associated with T1D susceptibility, contributing 10% of genetic susceptibility to T1D. Over the last decade, whole genome screens have identified at least 40 other loci associated with T1D. Furthermore, mutations in genes found in several of the susceptibility loci, such as *il-2*, *il-2ra*, *Ctla-4*, *PTPN22*, *il-10* have various autoimmune manifestations, including T1D. These genes are associated with the regulation of immune responses either by intrinsically controlling T and B cell reactivity or by enhancing the development and homeostasis of immunosuppression mediated by regulatory T (Treg) cells expressing Foxp3, a DNA-binding forkhead winged helix transcriptional regulator known to drive their lineage development.

Genetic susceptibility in humans and mice are both linked to variations in the IL-2 signaling pathway. In humans, T1D risk is related to the gene region encoding IL-2Rα, whereas in NOD mice the IL-2 gene (*Idd3* locus) confers susceptibility (Lyons et al., 2000). Additionally, it has been established that phosphorylation of STAT5, a crucial IL-2 signaling molecule, is reduced in T1D patients, and could account for diminished Treg cell numbers (Long et al., 2010). Furthermore, proper IL-2 signaling is essential for protection from diabetes in NOD mice. Work from our group and others has demonstrated that protective IL-2 allelic variants favor the expansion and suppressive function of Treg cells directly in the islets (Sgouroudis et al., 2008). Moreover, treatment of diabetic mice with IL-2 increases Treg cell numbers and induces expression of Treg cell-associated proteins such as Foxp3, CD25, ICOS, and CTLA-4. Collectively IL-2 preferentially enhances Treg immunosuppression and down-regulates IFNy production by pathogenic, islet-infiltrating effector T cells (Teff) (Grinberg-Bleyer et al., 2010).

## Foxp3+ Treg Cells – Master Regulators of the Immune System

**Figure 2:**
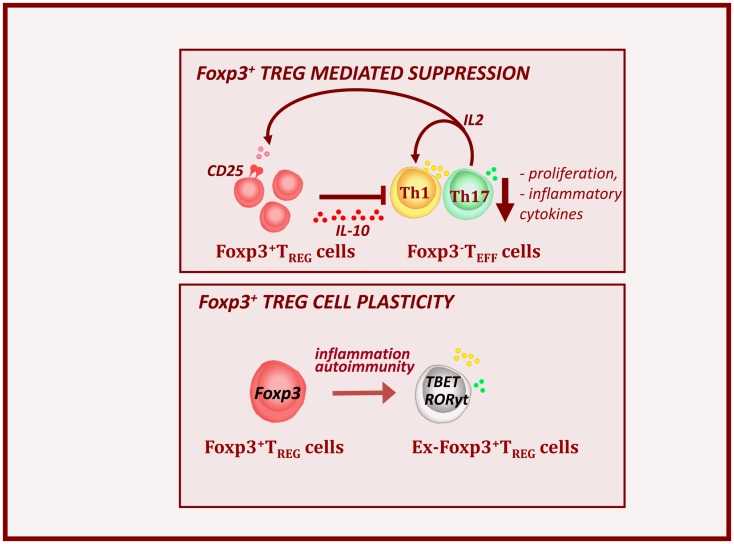
**Foxp3+ Treg cells control autoimmunity and T1D pathogenesis**. Foxp3+ Treg cells express high levels of IL-2Ra or CD25 and are dependent on Teff-derived IL-2. Alterations in local IL-2 production may precipitate T1D by perturbing Treg cell function. Treg cells produce the immunosuppressive cytokine IL-10. This results to the down-regulation of inflammatory cytokines (IFNy and IL-17) and decreased expansion of effector cell pools. However during the T1D progression, inflammatory signals, can provoke loss of Foxp3 expression. These, so called ex-Foxp3 Treg cells acquire an effector cell phenotype in terms of transcription factor expression and inflammatory cytokine production and contribute to T1D pathology. As widely observed features of Treg cell biology in autoimmune.

Natural CD4^+^ Treg cells which express Foxp3 and develop in the thymus, represent a unique lineage of T cells with the ability to suppress autoimmune and pathological responses (Figure 2, top panel) (Piccirillo et al., 2005). They represent 1–10% of thymic and peripheral circulating CD4^+^ T cells in mouse and human, and are able to down-regulate the activation and function of various immune effector cell subsets. Alternatively, Treg cells can differentiate in the periphery from conventional T cells upon reception of antigen-specific stimulation along with tolerogenic cytokine signals. Natural and induced Treg cells are characterized by the constitutive expression of the IL-2Rα chain (CD25) and preferentially express Foxp3 (Fontenot et al., 2005). The importance of Foxp3 has been demonstrated by mutations in the *foxp3* gene that result in the loss of Treg cell function and the development of multi-organ autoimmunity, including autoimmune diabetes, in IPEX patients and Scurfy mice (Hori et al., 2003; d’Hennezel et al., 2012). Several factors like IL-2 and TGFβ have been identified that can enhance stabilize Foxp3 expression via demethylation of CpG motifs within conserved regions of Foxp3 promoter (Shen et al., 2009; Haiqi et al., 2011). Treg cell mediated suppression mechanisms are numerous and complex, including several cell surface and soluble factors that directly control activation of effector cells. Suppression is likely mediated via cell–cell contact dependent mechanisms and production of immunomodulatory cytokines such as IL-10 and TGF-β, IL-35, that inhibit DC and T cell activity (Sakaguchi et al., 2009; Shevach, 2009).

## The Immune-Protective Role of Treg Cells in T1D

The critical importance of Treg cells in autoimmune settings, such as T1D, is well established (Atkinson and Leiter, 1999; Bach and Chatenoud, 2001). In NOD mice, depletion of CD4+CD25+ Treg cells accelerates development of T1D (Salomon et al., 2000; Salomon and Bluestone, 2001). Furthermore, abolishment of co-stimulatory pathways that are vital for Treg homeostasis, such as CD28 and ICOS, in NOD mice exacerbates T1D (Salomon and Bluestone, 2001; Anderson and Bluestone, 2005; Kornete et al., 2012).

We and others have shown that T1D progression is NOD mice is associated with a decrease in numbers and function of Treg cells in the inflamed islets and defects in IL-2 production by effector T cells seem largely responsible (Sgouroudis et al., 2008; Tang et al., 2008; Tritt et al., 2008; Kornete et al., 2012). Overall, this demonstrates that Treg cells function as major controllers of immune homeostasis and tolerance in the periphery. However, more recent finding indicated that Treg cells can become unstable and lose Foxp3 expression in inflamed pancreatic sites during T1D progression (Figure 2, bottom panel) (Zhou et al., 2009; McClymont et al., 2011). In the NOD mice, the lack of intra-islet IL-2 results in CD25 down-regulation and consequent reduction in Foxp3 expression. These ex-Foxp3+ T cells produce inflammatory cytokines, such as IFNy and IL-17 and have increased immunopathogenic potential upon adoptive transfer (Zhou et al., 2009). A similar observation has been made in T1D patients, in which a significant increase in the number of IFNγ and IL-17 secreting Foxp3+ Treg cells was observed (McClymont et al., 2011).

## Beta (β) Cells: Regeneration and Trans-Differentiation

The presence of β-cells in patients with long lasting T1D, despite ongoing autoimmunity, suggests that regeneration of β-cells may occur. About 60% of T1D patients enter a clinical “honeymoon phase,” lasting between 3 months and 2 years, and characterized by improved insulin secretion to the extent that some patients can discontinue exogenous insulin (Muhammad et al., 1999; Abdul-Rasoul et al., 2006). β-cells have a robust capacity to regenerate by proliferation, likely in response to inflammation-driven signals (Akirav et al., 2008). Furthermore, recent studies revealed a previously unrecognized plasticity of endocrine cells in the pancreas. However, depending on the nature of experimental β-cell destruction, these studies reached divergent conclusions regarding the origin of new β-cells. Supporting the hypothesis of self-renewal, partial abolition results in β-cell regeneration from surviving β-cells (Dor et al., 2004; Nir et al., 2007). However, when more>99% of β-cells are chemically destroyed, new β-cells are generated either via differentiation of endocrine and islet precursors (Guz et al., 2001; Thyssen et al., 2006) or by spontaneous reprograming of differentiated endocrine cell types such as δ-cells (Fernandes et al., 1997) or α-cells (Chung et al., 2010; Thorel et al., 2010; Zaret and White, 2010).

Several factors have been proposed to influence β-cells regeneration. Inflammation, though driving autoimmune responses, is implicated in β-cells proliferation. For example, while Th1 cell-derived IFN-γ is the main mediator of diabetogenesis in the NOD mice, the transgenic expression of IFN-γ enhances β-cells proliferation and survival (Tuch et al., 1991; Ablamunits et al., 2007). Similarly, inflammatory cytokines such as IL-1β, nitric oxide (NO), and TNF-α were shown to cause β-cell replication (Donath et al., 2003; Luo et al., 2005). More recently, Herold’s group demonstrated that increased β-cell proliferation depends on the inflammatory infiltrate itself and immunotherapeutic regimens, namely administration of anti-CD3 mAb and Foxp3^+^ Treg cells that suppress inflammatory T cells, also decrease β-cell replication (Sherry et al., 2006).

## The Control of T1D Complications by Treg Cells: The Case for Atherosclerosis

Persistent, dysregulated inflammation contributes to the development of secondary chronic disorders such as vascular or neurodegenerative disease in T1D patients. One of the most common T1D complications is atherosclerosis, an inflammatory disorder of the arterial wall caused by the retention of cholesterol in the sub-endothelial region of the artery. Whereas inflammation is driven by both innate and adaptive immune effector cells, recent studies suggest that Foxp3+ Treg cells control the development and progression of atherosclerosis (Veillard et al., 2004; de Boer et al., 2007). Treg cells account for 1–5% of T cell population within atherosclerotic lesions in which they produce of immunomodulatory cytokines, such as IL-10 and TGF-β (de Boer et al., 2007). Pro-atherogenic ApoE-deficient mice exhibit significantly lower numbers of Treg cells than their wild type counterparts (Mallat et al., 2003). The role of Treg cells was further elucidated via generation of low density lipoprotein receptor knock-out chimeric mice (Ait-Oufella et al., 2006). Reconstitution of these mice following irradiation with CD80/CD86 and CD28 deficient bone marrow cells resulted in a marked reduction in Treg cells and an increase in atherosclerotic lesion size compared to control mice (Ait-Oufella et al., 2006). Finally, various successful immune therapies used in atherosclerosis suggest an important role for Treg cells. Use of anti-CD3 mAB reduced plaque formation when administered prior to a high cholesterol diet and markedly decreased lesion progression in mice with established atherosclerosis (Steffens et al., 2006). Vaccine administration into atherosclerotic prone mice of pro-atherogenic auto-antigens, such as apolipoprotein B-100 or heat shock protein, led to the inhibition of atherosclerosis development. Both treatments resulted in increased production of Foxp3+ Treg cells and secretion of TGF-β and IL-10 (van Puijvelde et al., 2006; Klingenberg et al., 2010).

## The Patho-Physiology of Type 2 Diabetes and Its Complications

Whereas the autoimmune etiology of T1D pathogenesis is well established, T2D was historically considered a non-immune condition. However, recent work highlighting adiposity-associated chronic inflammation in T2D implicates immune mediators in metabolic dysregulation. In conjunction with adipocytes, the innate and adaptive immune system drives systemic inflammation, promoting both insulin resistance and associated complications such as diabetic nephropathy (DN). As crucial mediators of peripheral tolerance, it is not surprising that within this environment Treg cells are key regulators of adipose tissue inflammation and resultant diabetogenesis.

Excess adiposity is associated with an increase in serum C-reactive protein (CRP) and pro-inflammatory cytokines such as IL-6 in humans (Visser et al., 1999). Furthermore, insulin resistance positively correlated with the levels of these cytokines in the blood of T2D patients (Bruun et al., 2003). The mechanism by which inflammatory mediators can disrupt intracellular metabolic signaling has been elucidated in mouse models. Stimulation of the JNK and NF-κB pathways by pro-inflammatory cytokines activates negative regulators of the insulin receptor pathway. Specifically JNK and IKK-β phosphorylate insulin receptor substrate 1 (IRS1) inhibiting tyrosine phosphorylation by the insulin receptor (Arkan et al., 2005; Sabio et al., 2010). Genetic ablation of these kinases results in significant amelioration of insulin resistance. Thus cross talk between insulin receptor and inflammatory signaling cascades can disrupt cell metabolism and exacerbate insulin resistance.

In addition to the predominant adipocyte population, lean adipose tissue contains an appreciable number of leukocytes in the absence of inflammation, suggesting involvement in fat tissue homeostasis (Lumeng et al., 2007; Feuerer et al., 2009). However, profound accumulation of inflammatory immune infiltrates accompanies the accrual of lipids in the visceral adipose tissue (mVAT) of obese mice (Weisberg et al., 2003; Nishimura et al., 2009). Most notably, macrophages of the inflammatory M1 subtype predominate in obese VAT over tolerogenic M2 macrophages found in lean fat (Lumeng et al., 2007). The M1 phenotype is the major source of pro-inflammatory cytokines that promote insulin resistance in adipocytes. Though in humans a dichotomous M1/M2 paradigm is absent, macrophages nonetheless accumulate in obese fat and drive inflammation (Zeyda et al., 2007). Recently, CD8^+^ Teff cells have been identified as key regulators of macrophage recruitment and switch to the M1 type in obese adipose tissue (Nishimura et al., 2009). CD8^+^ cells were observed prior to macrophage populations in VAT of mice when obesity was induced via high fat diet (HFD). Furthermore, in CD8 depleted or deficient mice on HFD, VAT macrophage infiltration and phenotypic switch was repressed and indices of metabolic dysfunction significantly ameliorated. Others have proposed that CD4^+^T_h_1 cells coordinate adipose tissue inflammation, describing restored insulin sensitivity via T_h_1 cell depletion in a HFD mouse model of obesity (Winer et al., 2009). Collectively, these studies posit that T lymphocytes drive adipose tissue macrophage (ATM) recruitment and differentiation and consequent chronic inflammation in T2D.

In contrast, T lymphocytes can also exact essential regulatory function in adipose tissue. In both mouse and human, Foxp3^+^ Treg cells have been found in both VAT and subcutaneous fat (SAT) (Feuerer et al., 2009; Eller et al., 2011; Zeyda et al., 2011). Indeed Treg cells comprised more than half of the total CD4 compartment in VAT of healthy C57BL/6 mice (Feuerer et al., 2009). These Treg cells produce high quantities of the anti-inflammatory cytokine IL-10 and uniquely express nuclear receptor PPAR-γ, which is necessary for their homeostasis and regulatory function in VAT (Feuerer et al., 2009; Cipolletta et al., 2012). Gene expression profiling indicates PPAR-γ and Foxp3 may coordinately regulate VAT Treg transcriptional programs necessary to suppress adipose-associated inflammation. This could be akin to how recently identified Treg subsets are phenotypically specified to suppress T_h_1, T_h_2, or T_h_17 responses (Campbell and Koch, 2011). While in both genetic and diet-induced mouse models a waning of VAT Treg cells accompanies increasing adiposity, the effect of obesity on VAT Treg cell numbers in humans remains controversial (Feuerer et al., 2009). Whereas some groups have found FOXP3 expression to negatively correlate with BMI, others report no change or an increase in FOXP3 in the fat of obese individuals compared to normal BMI controls (Feuerer et al., 2009; Eller et al., 2011; Zeyda et al., 2011). However, these studies indirectly enumerate Treg cells via qPCR rather than flow cytometry and thus do not discriminate between FOXP3 expression by *bona fide* Treg cells, and transient FOXP3 upregulation by Teff cells upon activation. Nevertheless, such findings highlight the risk in literal application of established mouse models to human T2D.

Thus T cells direct adipose-induced systemic inflammation and therefore may indirectly promote T2D complications via metabolic disruption. Further evidence suggests T cells could mediate immunopathology directly in the target organ. For instance, profound infiltration of the kidneys by activated T cells is associated with the development of DN in T2D patients (Moon et al., 2012). This is accompanied by macrophage accumulation and production of pro-inflammatory cytokines such as IFN-γ and IL-1β (Galkina and Ley, 2006). However the relative contribution of inflammatory verses metabolic and hemodynamic factors to the initiation and progression of renal lesions remains unclear. A study of DN in db/db mice suggests Treg cells may dampen kidney immunopathology (Eller et al., 2011). Treg depletion via administration of anti-CD25 antibodies exacerbated nephropathy, renal dysfunction and leukocyte infiltration of the kidneys. Furthermore, adoptive transfer of Treg cells into db/db mice improved kidney function and ameliorated DN. Thus, Treg cells suppress inflammation both at the primary and secondary sites of T2D pathogenesis. Like in T1D, Treg cells may represent a key homeostatic checkpoint that, if breached, results in the breakdown of peripheral tolerance and progression of autoreactive responses.

## Immunotherapeutic Strategies: Current and Future Avenues

Type 1 diabetes susceptibility and pathogenesis results from a complex interplay between genetic, environmental, and immunological factors. Therefore, a multi-faceted solution is likely necessary to effectively treat T1D. An ideal immunotherapy would simultaneously shut down pathogenic T cells and enhance regulatory mechanisms, while also promoting β-cell regeneration or neogenesis. All three therapeutic goals have been proposed to occur through anti-CD3 mAB therapy. Anti-CD3 works as an immune suppressant, promotes antigen-specific Treg cells and both increases and preserves β-cell mass. Anti-CD3 mAB causes internalization of the CD3-TCR complex and prevents Teff cells from recognizing antigen. Furthermore it affects TCR-mediated signal transduction and provokes apoptosis and anergy of Teff cells (Chatenoud et al., 1982, 1994). Beyond modulation of effectors cell pools, anti-CD3 has recently been shown to promote induction and stabilization of Treg cells (You et al., 2007; Penaranda et al., 2011). Two independent clinical trials using either Teplizumab (United States) or Otelixizumab (Europe) led to the sustained preservation of insulin production. In NOD mice, anti-CD3 therapy permanently reversed diabetes and in humans C-peptide levels were sustained from 1 to 5 years, demonstrating long term protection could be obtained (Herold et al., 2002, 2009; Keymeulen et al., 2005).

Can antigen-specific therapies prevent the immune-driven pathology in disease? In T1D, several studies had focused on the use of insulin and GAD65 as a primary targets for antigen-specific therapies as they are proposed to be key initiating auto-antigens in NOD mice and major auto-antigens in human. The most prominent clinical trial so far involves oral insulin administration in first-degree relatives of T1D patients with high levels of insulin autoantibodies. This regimen modulates diabetogenic immune responses and consequently delays diabetes onset by as much as 5 years (Skyler et al., 2005). In addition, a single injection of the GAD-alum vaccine, the most successful antigen-specific therapy to date, delayed the loss of C-peptide production in new onset T1D children and adolescents (Ludvigsson et al., 2008). Administration of agents such as gastrin that stimulate beta cell neogenesis without increasing proliferation, can minimize antigen spread and prevent β-cell loss (Rooman et al., 2002). Lastly, modulation of local tissue or systemic metabolism, possibly by targeting the PPAR-γ pathway, may impact the generation of adipose-related Treg cells and suppress local inflammatory responses.

## Conclusion

Thus in T1D and T2D, inflammation in the target tissue and at secondary sites drives disease progression. Whereas in T1D this response is quintessentially autoimmune, the factors that initiate adipose tissue inflammation in T2D have yet to be elucidated. However, in both conditions innate and adaptive leukocyte infiltration and local tissue destruction instigate chronic systemic inflammation. This promotes diabetogenic complications including autoimmune responses at secondary tissues and metabolic perturbation in T2D. Treg cells, potent suppressors of autoimmunity in the periphery, can dampen immune effector cell responses in the β-islets. Furthermore, an important role for this subset in inflamed adipose tissue has recently been characterized. Thus, enhancing the activity of Treg cells may present a therapeutic avenue to limit type 1 and type 2 diabetes pathogenesis and its complications.

## Conflict of Interest Statement

The authors declare that the research was conducted in the absence of any commercial or financial relationships that could be construed as a potential conflict of interest.
